# Choice Under Risk: How Occupation Influences Preferences

**DOI:** 10.3389/fpsyg.2019.02003

**Published:** 2019-08-30

**Authors:** Tetiana Hill, Petko Kusev, Paul van Schaik

**Affiliations:** ^1^Hertfordshire Business School, University of Hertfordshire, Hatfield, United Kingdom; ^2^Huddersfield Business School, University of Huddersfield, Huddersfield, United Kingdom; ^3^School of Social Sciences, Humanities & Law, Teesside University, Middlesbrough, United Kingdom

**Keywords:** choice under risk, risk preferences, occupation, utility, decision context

## Abstract

In the last decade, a number of studies in the behavioral sciences, particularly in psychology and economics, have explored the complexity of individual risk behavior and its underlying factors. Most previous studies have examined the influences of various socio-economic, cognitive, biological and psychological factors on human decision-making, however, the relationship between the decision-makers’ risk preferences and occupational background has not received much empirical attention. Accordingly, in the current study, we investigated how occupational background, together with decision-making framing (e.g., variations in decision domain, context, presentation of risk, and utility ratios), influence participants’ risk preferences for decision options with equivalent expected utility. Our novel findings indicate that risk preferences may vary among individuals from different occupational backgrounds. As such, when the task was framed in gain terms, participants who mostly deal with health/safety-related risks on a day-to-day basis (*high-risk* occupations) were predominantly risk-averse (avoiding risky options), while participants who mostly deal with financial/social risks (*white-collar* occupations) were prone to risk-seeking behavior (avoiding certain options). Specifically, in “high-risk” occupations, participants’ pattern of choices changed from risk-averse in gain scenarios to risk-seeking in loss scenarios. However, the opposite pattern of risk preferences was found in participants with “white-collar” occupations. Our findings indicate that decision-makers’ occupational backgrounds influence risk preferences under some circumstances.

## Introduction

In the last decade, a number of studies have been conducted to explore the complexity of human choice (e.g., Kahneman and Tversky, 1979; Tversky and Kahneman, 1992; Slovic, 2000; Hertwig et al., 2004; Kusev et al., 2009). Nonetheless, scholars remain actively engaged in discussions on whether people’s preferences for a particular decision option should be considered as unitary or multidimensional (e.g., Slovic, 1964, 1987; Frey et al., 2017), and whether these preferences are relatively stable or may change over time (e.g., Josef et al., 2016; Frey et al., 2017; Kusev et al., 2017, 2019). According to normative theories (e.g., von Neumann and Morgenstern, 1947), human choice is made based on stable preferences and utility maximization strategies. This means that when making decisions, people are expected to (i) make rational decisions, and (ii) evaluate each of the decision options based on norms, rules and regulations in order to select the option with the highest expected value/utility. Based on the principles of normative theories, people’s preferences for a particular decision option should not be affected by a decision domain and/or different wordings of tasks. However, previous studies indicate that people tend to make decisions that violate the principles of normative models, mostly because they possess incomplete knowledge about the decision options, and have insufficient computational and cognitive skills, which altogether create constraints on people’s cognitive capabilities (Simon, 1956; Kahneman, 2011).

In comparison to normative theories, descriptive theories of decision making (e.g., Tversky and Kahneman, 1992) postulate that people’s decisions are based on computational processing, where the decision-making attributes are integrated into subjective expected values. These theories predominantly focus on the circumstances (both external and internal factors) under which a particular decision option was chosen. However, Kahneman (2011) suggests that preferences within certain circumstances can be consistent, meaning that if the various internal and external factors which can influence preferences are identified, it is possible to predict decision-making behavior. In order to identify these factors, more recent studies were conducted to understand the psychological mechanisms of choice based on non-computational processing and decision experience (e.g., Hertwig et al., 2004; Kusev et al., 2009; Newall and Love, 2015). In a review conducted by Kusev et al. (2017) based on the results of previous studies, the authors conclude that a number of factors, such as socio-economic, cognitive, biological and psychological factors, may affect people’s decision-making under risk. In addition, Kusev and van Schaik (2011) highlight that decision behavior is influenced by (i) decision-making context and content, (ii) individual differences of a decision maker (e.g., cognitive skills and motivation), and (iii) the way task material is presented (frequency/probability/domain). Although some previous studies were carried out to investigate the specifics of decision-making processes within various occupational domains such as health (e.g., van Schaik et al., 2005), business (e.g., Olsen, 1997), and finances (e.g., Porcelli and Delgado, 2009), only a few studies were conducted to compare risk preferences among people with different occupational backgrounds (e.g., Baron et al., 2000; Swait and Adamowicz, 2001). Moreover, psychometric studies of risk perception (e.g., Slovic, 1964, 1987) have revealed that (i) people with risk-related and non-risk-related occupations have different perceptions of risk (Grable, 2000), and (ii) their judgments of risk are predominantly affected by internal psychological factors rather than probabilities and expected utility of each course of actions (Slovic, 1964, 2000). Additionally, Grable (2000), while investigating the tendencies of financial risk-taking and tolerance with regard to demographics and socio-economic background, suggested that among several factors, such as gender, age, marital status, and educational background, job position and occupational background, are associated with choices that lead to greater financial achievements and success. Furthermore, LeBoeuf et al. (2010) suggest that occupation, together with gender, shape self-identity, which then informs expressed preferences and choice patterns. While investigating the role of worry, Baron et al. (2000) reported that experts in risk analysis and non-experts differ in their perception of particular risks mostly in what they consider as small/big risk. Similarly, research conducted by Masclet et al. (2009) showed that those employees, who are self-employed (or freelance occupations), are more risk-averse than salaried workers. To sum up, these studies provide empirical evidence, suggesting that in order to deepen knowledge on human choice and decision-making processes, risk preferences should be investigated, taking into account people’s occupational background.

### Occupational Background and Risk Preferences

Although people from all walks of life have to make decisions that involve various degrees of risk and uncertainty, it is plausible that their risk preferences may differ depending on their occupational backgrounds. For instance, stock-market brokers make on a day-to-day basis various decisions that include financial and social risks under extreme time constraints, while medical practitioners mostly deal with health- and safety-related risks, which may have substantial consequences to life and the well-being of other people (Kozena and Frantik, 2001). Therefore, people encounter different types of work-related risks posed by their occupational environments, which could also to a certain degree inform their risk preferences (Weber et al., 2002). For example, decision environments of occupations that predominantly pose financial and social risks to employees are quite often induced by risk-seeking expectations and where risk-taking willingness is normally rewarded. As such, most startup companies tend to employ new staff with a propensity to risk-taking behaviors when expanding their payroll (Weber et al., 2002). In contrast, those occupational environments that pose risks related to health and safety challenge people to systematically comprehend all the decision options and ideally compare them, as the consequences of their decisions could affect properties and lives. Accordingly, it is expected that people who deal with either health/safety or financial/social risks possess particular computational skills (Tversky and Kahneman, 1986) and a stable system of preferences (Kusev and van Schaik, 2011) which enable them to analyze choice alternatives, decision outcomes, and choose the most appropriate course of action. However, considering that people are prone to changing their decision preferences (Kahneman, 2011; Kusev et al., 2017), one of the aims of the current study is to examine whether and how risk preferences may vary for people from different occupational backgrounds, depending on the way the decision tasks are framed.

### The Framing Effect

It is well established that people respond inconsistently to decision-making problems with equal utilities that are *framed* in different ways. For example, McNeil et al. (1982) found that participants chose a surgery for the treatment of lung cancer over radiation therapy when the surgery outcome was framed positively in terms of survival rates rather than the probability of dying. This framing effect was clearly exhibited in Tversky and Kahneman’s (1981) seminal paper where participants were presented with an experimental hypothetical task to combat an Asian disease and asked to select one of two intervention programs (representing either a certain or a probabilistic option). When the task was framed in gain terms, the majority of participants chose the risk-averse (certain) program. However, when the task was framed in loss terms, most participants chose the risky (probabilistic) program. This empirical evidence showed that people violate the principles of rational choice, and these violations vary in the domains of loss and gain. As such, the results of this study suggest that (i) losses loom larger than corresponding gains (loss aversion), and (ii) small probabilities of risk are overweighted and large probabilities are underweighted, exemplifying risk-averse behavior in the domain of gain and risk-seeking behavior in the domain of loss (Tversky and Kahneman, 1992). Moreover, further research on the effect of extremeness aversion (Tversky and Kahneman, 1992; Tversky and Simonson, 1993) has revealed that choice options with extreme values (e.g., high and low probability levels) within an offered set are relatively less attractive than options with intermediate values. In an attempt to explain the framing effect and contributing factors to it, scholars have also highlighted the role of decision-making context and content in choice under risk (e.g., Kusev et al., 2009, 2017, 2019; Vlaev et al., 2010; Jones and Oaksford, 2011; Kusev and van Schaik, 2011; Reimann et al., 2014). Specifically, Kusev and van Schaik (2011) distinguish decision-making content from context by defining content as a memory representation of experienced events and context as a description and presentation of risk. Another study conducted by Kusev et al. (2009) also revealed that people’s experiences of events leak into decisions even when risk information is explicitly provided. Finally, scholars suggest that risky preferences may vary depending on framing domain (scenario) in which decisions are made, making it another factor contributing to the framing effect. Previous studies have also explored risky decision-making across various scenarios related to animals, health, money and even aliens (see Heilman and Miclea, 2016); however, a meta-analysis conducted by Kühberger (1998) revealed that risky preferences within scenarios related to human life and health are more affected by the framing effect than other scenarios. Given this evidence, it can be assumed that decision-making framing (variations in decision domain, context, presentation of risk, and utility ratios) and occupational background may influence decision strategies and preferences for risk (e.g., Rettinger and Hastie, 2001; Kusev et al., 2009).

It is plausible that occupational background facilitates learning of behavioral decision strategies, which influences risk preferences and dominates the sensitivity to contextually presented probabilistic information (e.g., Dayan and Daw, 2008; Vuckovic et al., 2013). Therefore, in this study, our aim is to investigate the influence of type of occupation and decision-making framing [e.g., variations in decision domain (loss and gain; Tversky and Kahneman, 1981), context, presentation of risk and utility ratios] on risk preferences. Taking into consideration occupational background of people, we anticipate that (i) those, who deal with financial/social risks posed by their occupational environment will be prone to risk-seeking behavior in the domain of gain (choosing the probabilistic option), and (ii) people who deal with health/safety-related risks will be prone to risk-averse preferences in the domain of gain (choosing the certain option). Given that there are a number of occupations that could be included in the study, we identified two groups of occupations based on what types of work-related risks they predominantly deal with, based on a classification of risks provided by Weber et al. (2002). The first group are those occupations that deal with health- and safety-related risks – *high-risk occupations*. The characteristic feature of this group is that a large proportion of tasks performed by workers can be described as safety-critical, meaning that possible consequences of performance error may lead to injury of workers or their co-workers, or members of the general public, and/or cause serious damage to the environment, production or equipment (Fan et al., 2016). Previous studies have also described this group of occupations as “safety-sensitive” and “safety-critical” (e.g., Hegmann et al., 2014; Hobson, 2019). Examples of occupations that were recruited to this group are police officer, medical worker (i.e., pharmacist, ambulance worker or surgeon), maintenance worker, and public transport driver. The second group are *white-collar* occupations, in which workers predominantly encounter financial and social risks and perform professional, managerial, or administrative work. Any performance errors committed by workers from this group of occupations can result in serious financial, legal, and corporate difficulties that may impact not just quality of products and services but also workers’ well-being and livelihoods (Fan et al., 2016). The consequences of these errors, however, do not cause the same adverse effects or pose direct health- or safety-related risks as in “high-risk” occupations. This group included financial consultants, sales executives, and investment bankers.

## Materials and Methods

### Participants

In total 120 participants (61 males and 59 females; age range: 22–71; mean age = 45; *SD* = 10.69) were recruited through a marketing company. Both occupational groups comprised equal number of participants: 60 in the “high-risk” group (32 males and 28 females; mean age = 46; *SD* = 10.42) and 60 in the “white-collar” group (29 males and 31 females; mean age = 45; *SD* = 10.85).

### Procedure

Before commencing with the recruitment of participants, a marketing company was provided with a list of occupations representing two groups (*high-risk* and *white-collar* occupations) to identify the appropriate individuals from their database. Upon completion of this step, a link with a survey was distributed among the participants by a marketing company. The participants were also informed that the study was voluntary and that their responses were anonymous and confidential. Participants took part individually and received a payment of £2.50. After giving consent, the participants were asked to make series of decisions in 32 trials (binary decisions). They were asked to consider each presented scenario in turn and each time choose one of two options. The participants were also informed that they were not under time pressure and each of the tasks was self-paced.

### Design and Materials

A mixed-measures 2 × (4) × (2) × (2) × (2) design was employed. The first between-subject variable was *occupation* (“high-risk” or “white-collar” occupations). The second repeated (within-subject) variable was *decision-making context* (health, ecology, technology, and finance). The third repeated variable was *utility ratio* derived from the presentation of two probability levels (high = 95%/5% and low = 66.7%/33.3%). The fourth repeated variable was *domain* of risky prospects (gain and loss). The fifth repeated variable was *presentation* of risk (textual and visual). The presentation order of levels of the independent variables was randomized. The dependent variable was *decision-making preference* [risk-averse, (preference for certain outcome) or risk seeking (probabilistic outcome)]. The instructions, scenarios and tasks were presented as part of an online computer-based experiment. Four decision contexts were developed [ecology (saving species), health (investigating an HIV treatment), technology (manufacturing mobile phones), and finance (stock investment), within two decision-making domains (loss and gain)]. *Utility ratio* was derived from the presentation of two probability levels (95%/5% and 66.7%/33.3%).

We used the Asian disease task developed by Tversky and Kahneman (1981) as a template for constructing decision scenarios, with a binary choice between a sure thing and a probabilistic outcome. All of the decision-making scenarios used in the experiment were hypothetical. Traditionally, in psychology and economics, the Asian Disease task is used to explore the influence of framing effects on risk preferences and “domain-specific” risk preferences (e.g., Kühberger, 1998; Levin et al., 1998). However, there is growing evidence suggesting that it can also be employed as a behavioral measure to reveal “true” risk preferences (e.g., Appelt et al., 2011; Schonberg et al., 2011; Frey et al., 2017). The key benefit of using this task is that it helps to capture specific cognitive processes, such as integration of losses and gains, which in turn underlines risk preferences. This informed our decision to choose this measure in the study to elicit “revealed” risk-taking behavior under various circumstances. Therefore, choice options of the task appeared for participants in text (as in the original Asian disease task) and visual forms. The visual presentation of choice was constructed as two pie charts, with the regions of the first pie chart showing the sure option and the regions of the second showing the probabilities (risk) for both gain and loss. An example of a scenario with a textual presentation of risk and health context with options for gain is as follows (see Supplementary Materials for the full list of the instructions, scenarios and tasks used in the study):

You are working in a laboratory which is inventing a medicine that will help to stop the spread of HIV. As a result of conducting an experiment with the new medicine, 600 people might die. You have come up with two alternative programs to lessen the harm caused by the medicines. Choose one of the following programs:

**Program A:** if Program A is adopted, 200 people will be saved.
**Program B:** if Program B is adopted, there is 33.3% probability that 600 people will be saved and 66.7% probability that no people will be saved.

## Results

**FIGURE 1:**
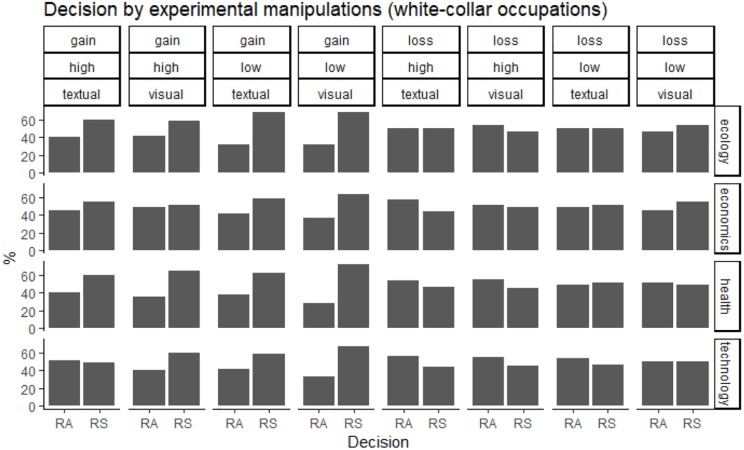
Decision in “white-collar” occupations, as a function of domain, utility ratio, presentation, and context (risk-averse preferences: RA; risk-seeking preferences: RS).

**FIGURE 2:**
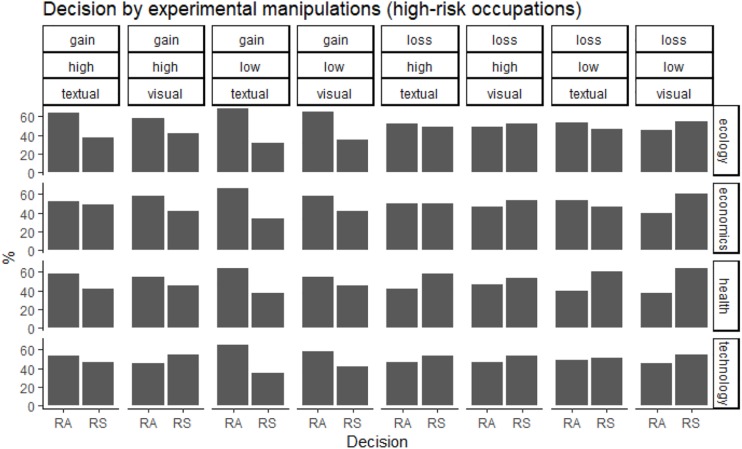
Decision in “high-risk” occupations, as a function of domain, utility ratio, presentation, and context (risk-averse preferences: RA; risk-seeking preferences: RS).

**FIGURE 3:**
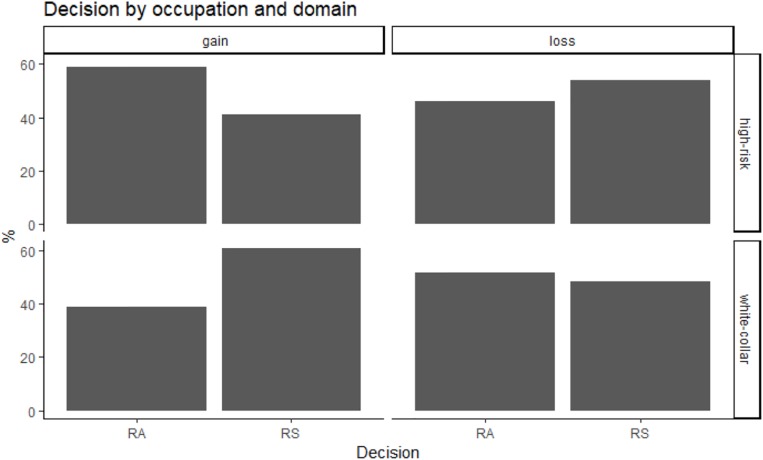
Decision as a function of occupation and domain (risk-averse preferences: RA; risk-seeking preferences: RS).

**FIGURE 4:**
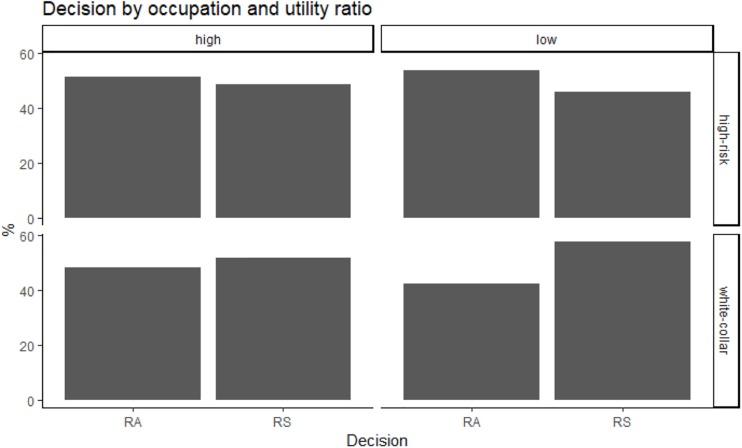
Decision as a function of occupation and utility ratio (risk-averse preferences: RA; risk-seeking preferences: RS).

**FIGURE 5:**
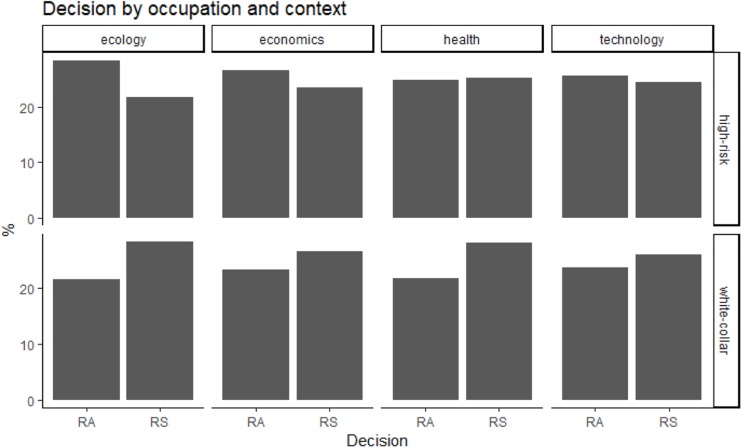
Decision as a function of domain and context (risk-averse preferences: RA; risk-seeking preferences: RS).

Overall, participants with “high-risk” occupations were more risk-averse (53% of choices for the certain outcome) than participants with “white-collar” occupations (45% of choices for the certain outcome; see also Figures 1, 2). Because the independent variables (domain, utility ratio, probability, presentation of risk and decision-making context) were used with repeated measures, the data were analyzed with multilevel modeling. In staged model-testing (recommended by Tabachnick and Fidell, 2013), the difference between subsequent models was tested. All models used a random intercept and fixed coefficients for the independent variables. The model with all main effects was statistically significantly different from the null model with no effects [chi square (7) = 14.18, *p* = 0.048]. A model with all main effects and all two-way interactions was significantly different from an all-main effects model [chi square (17) = 108.07, *p* = 2.78 × 10^–15^]. Models with all three-way, four-way and five-way interactions were not significantly different from models with all two-way, three-way, and four-way interactions, respectively. The significant two-way interactions were occupation by domain (*z* = −9.18, *p* < 2 × 10^–16^), occupation by utility ratio (*z* = 3.14, *p* = 0.002), occupation by decision-making context, specifically in health versus ecology context (*z* = −2.05, *p* = 0.04), and technology versus ecology contexts (*z* = −2.68, *p* = 0.007). In particular, in “high-risk” occupations, the pattern of choices changed from risk-averse in gain scenarios to risk-seeking in loss scenarios, but the opposite was true in “white-collar” occupations (Figure 3). “High-risk” occupations were more risk-averse than “white-collar” occupations, but this difference increased from a high utility ratio to a low utility ratio (Figure 4). Moreover, “high-risk” occupations were more risk-averse than “white-collar” occupations in ecology scenarios, but the difference was smaller in health and technology scenarios (Figure 5).

Because occupation was involved in all significant interaction effects and to reflect the focus on the variable occupation, a follow-up analysis was conducted separately for “high-risk” and “white-collar” occupations.

### “White-Collar” Occupations

As before, in staged model-testing, the difference between subsequent models was tested. The all-main effects model was significantly different from the null model,^1^ but none of the higher-order models were significantly different from its next-lower-order model. In the all-main effects model, the effects of domain and utility ratio were significant (Table 1). The odds of risk-seeking were greater with losses and low utility ratios.

**TABLE 1:** Regression coefficients, “white-collar” occupations.

	**95% Confidence interval**		
	**OR**	**Lower limit**	**Upper limit**
(Intercept)	1.69	1.07	2.72
Domain (loss)	0.51	0.41	0.62
Presentation (visual)	1.16	0.94	1.43
Utility ratio (low)	1.39	1.13	1.72
Context (economics vs. ecology)	0.82	0.61	1.11
Context (health vs. ecology)	0.97	0.72	1.30
Context (technology vs. ecology)	0.78	0.58	1.05

### “High-Risk” Occupations

As before, in staged model-testing, the difference between subsequent models was tested. The all-main effects model was significantly different from the null model,^2^ but none of the higher-order models were significantly different from its next-lower-order model. In the all-main effects model, the effects of domain, presentation, context (health versus ecology), and context (ecology versus technology) were significant (Table 2). The odds of risk-seeking were greater with gains, with visual presentation, in economics scenarios (in comparison with ecology scenarios) and in technology scenarios (in comparison with ecology scenarios).

**TABLE 2:** Regression coefficients, “high-risk” occupations.

	**95% Confidence interval**		
	**OR**	**Lower limit**	**Upper limit**
(Intercept)	0.45	0.27	0.74
Domain (loss)	2.08	1.67	2.59
Presentation (visual)	1.27	1.03	1.58
Utility ratio (low)	0.86	0.70	1.07
Context (economics vs. ecology)	1.23	0.91	1.67
Context (health vs. ecology)	1.51	1.11	2.05
Context (technology vs. ecology)	1.39	1.02	1.88

## Discussion

In the present study, using the Asian disease task, our aim was to examine whether and how occupational background and decision-making framing (e.g., variations in decision domain, context, presentation of risk, and utility ratios) influence preferences for certain or probabilistic options with equivalent risk information. Overall, our findings suggest that occupational background influences people’s risk preferences, specifically, participants with “high-risk” occupations were predominantly risk-averse, while participants with “white-collar” occupations were mostly risk-seeking even after decision outcome values and probabilities were known to them. Specifically, in “high-risk” occupations, the pattern of preferences changed from risk-averse in gain scenarios to risk-seeking in loss scenarios, while the opposite pattern of risk preferences was found in “white-collar” occupations. In contrast to previous studies (e.g., von Neumann and Morgenstern, 1947; Tversky and Kahneman, 1981, 1992), we hypothesized that the patterns of risk preferences may vary within two groups of occupational profiles.

Although previous studies indicate that risk preferences are generic and stable psychological patterns (e.g., Tversky and Kahneman, 1981, 1992; Tversky and Simonson, 1993), our results show that participants with “white-collar” occupations exhibited relatively more risk-seeking preferences in the domain of gain (choosing the probabilistic option). The opposite behavioral pattern, however, was observed in participants with “high-risk” occupations as they exhibited relatively more risk-averse preferences in the domain of gain (choosing the certain option). These findings generally contradict the prospect theory that predicts an overall risk-averse behavior for gains and risk-seeking behavior for losses in general population (Tversky and Kahneman, 1981, 1992). Our findings could be explained in several ways. Firstly, people differ in their risk perceptions and attitudes toward perceived risk and therefore may not only choose specific occupations with regards to these attitudes, but also be selected by organizations for a particular job based on a match in risk attitudes (Weber et al., 2002; Bonin et al., 2007). According to Bonin et al. (2007), the more individuals are willing to take risks, the more likely they are to end up working in an occupation where a risk-taking propensity is rewarded. Secondly, people may choose occupations based on their sensation-seeking propensity. In particular, the results of the study conducted by Wong and Carducci (1991) show that individuals who deal with financial risks on a day-to-day basis score higher on the scale of sensation-seeking and overall are more willing to take risks. The authors also conclude that these individuals tend to choose occupations that would include a certain degree of change, flexibility, and risk. Future research should further explore the associations between personality traits, occupational background and risk preferences. Lastly, these findings could be explained in terms of the reinforcement (Dayan and Daw, 2008) and adaptive-learning theories (Hertwig and Erev, 2009; Vuckovic et al., 2013). These theories suggest that people may learn to prefer a particular option to an alternative option with identical expected value as a result of working within a particular occupational environment. This may also suggest that risk preferences can be formed by experience and types of work-related risks people encounter.

The results also revealed that participants with “high-risk” occupations in the domain of gain were more risk-averse than participants with “white-collar” occupations, but this difference increased from a high utility ratio to a low utility ratio. These participants exhibited relatively more risk-averse preferences for options with low and high utility ratios (uncertainty aversion). In contrast, participants with “white-collar” occupations in the domain of gain were predominantly risk-seeking for options with low and high utility ratios (certainty aversion). This difference in choice preference between the two types of occupational backgrounds was particularly evident for the low utility ratio (both choices with intermediate probabilities) compared to the high utility ratio (both choice options with extreme probabilities – high versus low). We also found that for participants with “white-collar” occupations, the odds of risk-seeking were greater with losses and low utility ratios (certainty aversion). Moreover, for participants with “high-risk” occupations, the odds of risk-seeking were greater with gains with visual presentation, in economics scenarios (in comparison with ecology scenarios) and in technology scenarios (in comparison with ecology scenarios). It is plausible that learnt behavioral strategies within specific occupations (expected risk aversion for high-risk occupations and expected risk-seeking for “white-collar” occupations) were employed by the participants in their decisions (e.g., Dayan and Daw, 2008; Vuckovic et al., 2013). It should be noted, however, that the study included only one decisional scenario per decision making context, which suggests that future research should further investigate the effect of occupational background on risk preferences using different tasks/scenarios per decision-making context.

In summary, our findings demonstrate that decision-makers’ occupational background may influence risk preferences under some circumstances. Both occupational groups that were recruited in our study, “high-risk” and “white-collar” occupations, experience and assess risk regularly whilst following pre-designed normative rules and regulations (Raju et al., 1995). However, both occupational groups deal with different types of work-related risks that could cause the variations observed in our study results, alongside other factors. Our findings provide more evidence toward a common assumption that risk preferences are not stable traits and may be influenced by the occupational background of decision-makers, which could be taken into a consideration in the construction and development of occupational risk-training and occupational selection.

Our study has some limitations. Firstly, the proposed categorization of occupational backgrounds as “high-risk” and “white-collar” (depending on what type of work-related risks people encounter) can be considered as hypothetical, rather than evidence-based. However, the fact that our results showed significant differences between these two types of occupations in terms of risk preferences provides further credibility for this categorization. Secondly, the methodology we employed in the study did not include any other methods of measuring risk preferences in addition to the Asian disease task, such as self-assessment scales, which are commonly used in other studies. However, it should be noted that while self-assessment scales are normally used to measure a risk-taking propensity, which is usually referred to as a “general trait,” we wanted to capture true risk-taking behavior. Furthermore, according to Frey et al. (2017) comparison of the reliability and validity of multiple risk-taking measures, this type of task is an appropriate behavioral measurement to elicit revealed risk preferences. Lastly, the study did not measure potential confounding variables that could influence the results, such as pay schemes, job seniority, years of experience of working within a particular occupation, as well as various personality traits which are commonly associated with a propensity to risk-taking. Future studies should address these limitations by taking into a consideration these variables when exploring the influence of occupational backgrounds on risk preferences.

## Ethics Statement

Ethics approval was granted by the Kingston University London, United Kingdom. The participants completed the online questionnaire distributed using Qualtrics. The first page of the questionnaire contained information regarding the nature and purpose of the study; why the participants have been specifically invited; what participation involves; that taking part is voluntary and they can withdraw from the study at any time with no consequences or penalty; how the collected data will be treated and that they complete the questionnaires completely anonymously. The participants were then asked to click on the appropriate tabs to signal that they agree/decline to participate in the study. Once they had clicked to agree to take part in the study, they would be directed to the questionnaire.

## Author Contributions

TH was responsible for drafting the manuscript, collecting the data, working on the final version, and the final corrections. PK was responsible for consulting the first draft and working on the final version together with TH. PK and PS analyzed the data. All authors approved the final version of the manuscript for submission.

## Conflict of Interest Statement

The authors declare that the research was conducted in the absence of any commercial or financial relationships that could be construed as a potential conflict of interest.

## Footnotes

1 The all-main effects model with the addition of age as a covariate was not significantly different from the model without the covariate.

2 The all-main effects model with the addition of age as a covariate was not significantly different from the model without the covariate.
