# Supplementary material for: Choice Under Risk: How Occupation Influences Preferences
Source: Front Psychol. 2019 Aug 30;10:2003. doi: 10.3389/fpsyg.2019.02003 (PMC6730483; doi:10.3389/fpsyg.2019.02003)
Supplement: Supplementary file 1 [file Table_1.DOCX]

Supplementary materials

1. **Decision-making tasks**

*Ecology domain*

You are the head of a manufacturing company and you need to cut down a large area of ​​trees. As a result, 600 species might die. You have come up with two alternative programs to lessen the harm caused by the company. Choose one of the following programs:

**Program A:** If Program A is adopted, 200 species will be saved.

**Program B:** If Program B is adopted, there is 33.3% probability that 600 species will be saved and 66.7% probability that no species will be saved.

You are the head of a manufacturing company and you need to cut down a large area of ​​trees. As a result, 600 species might die. You have come up with two alternative programs to lessen the harm caused by the company. Choose one of the following programs:

**Program A:** If Program A is adopted, 400 species will die.

**Program B:** If Program B is adopted, there is 33.3% probability that no species will die and 66.7% probability that 600 species will die.

You are the head of a manufacturing company and you need to cut down a large area of ​​trees. As a result, 600 species might die. You have come up with two alternative programs to lessen the harm caused by the company. Choose one of the following programs:

**Program A:** If Program A is adopted, 30 species will be saved.

**Program B:** If Program B is adopted, there is 5% probability that 600 species will be saved and 95% probability that no species will be saved.

You are the head of a manufacturing company and you need to cut down a large area of ​​trees. As a result, 600 species might die. You have come up with two alternative programs to lessen the harm caused by the company. Choose one of the following programs:

**Program A:** If Program A is adopted, 570 species will die.

**Program B**: If Program B is adopted, there is 5% probability that no species will die and 95% probability that 600 species will die.

You are the head of a manufacturing company and you need to cut down a large area of ​​trees. As a result, 600 species might die. You have come up with two alternative programs to lessen the harm caused by the company. Choose one of the following programs:

**Program A:** If Program A is adopted, some species represented in black will be saved (see the pie-chart below).

**Program B:** If Program B is adopted, the probability that 600 species will be saved is represented in black and the probability that no species will be saved is represented in grey (see the pie-chart below).

You are the head of a manufacturing company and you need to cut down a large area of ​​trees. As a result, 600 species might die. You have come up with two alternative programs to lessen the harm caused by the company. Choose one of the following programs:

**Program A:** If Program A is adopted, some species represented in grey will die (see the pie-chart below).

**Program B:** If Program B is adopted, the probability that no species will die is represented in black and the probability that 600 species will die is represented in grey (see the pie-chart below).

You are the head of a manufacturing company and you need to cut down a large area of ​​trees. As a result, 600 species might die. You have come up with two alternative programs to lessen the harm caused by the company. Choose one of the following programs:

**Program A:** If Program A is adopted, some species represented in black will be saved (see the pie-chart below).

**Program B:** If Program B is adopted, the probability that 600 species will be saved is represented in black and the probability that no species will be saved is represented in grey (see the pie-chart below).

You are the head of a manufacturing company and you need to cut down a large area of ​​trees. As a result, 600 species might die. You have come up with two alternative programs to lessen the harm caused by the company. Choose one of the following programs:

**Program A:** If Program A is adopted, some species represented in grey will die (see the pie-chart below).

**Program B:** If Program B is adopted, the probability that no species will die is represented in black and the probability that 600 species will die is represented in grey (see the pie-chart below).

*Health domain*

You are working in a laboratory which is inventing a medicine that will help to stop the spread of HIV. As a result of conducting an experiment with the new medicine, 600 people might die. You have come up with two alternative programs to lessen the harm caused by the medicines. Choose one of the following programs:

**Program A:** If Program A is adopted, 200 people will be saved.

**Program B:** If Program B is adopted, there is 33.3% probability that 600 people will be saved and 66.7% probability that no people will be saved.

You are working in a laboratory which is inventing a medicine that will help to stop the spread of HIV. As a result of conducting an experiment with the new medicine, 600 people might die. You have come up with two alternative programs to lessen the harm caused by the medicines. Choose one of the following programs:

**Program A:** If Program A is adopted, 400 people will die.

**Program B:** If Program B is adopted, there is 33.3% probability that no people will die and 66.7% probability that 600 people will die.

You are working in a laboratory which is inventing a medicine that will help to stop the spread of HIV. As a result of conducting an experiment with the new medicine, 600 people might die. You have come up with two alternative programs to lessen the harm caused by the medicines. Choose one of the following programs:

**Program A:** If Program A is adopted, 30 people will be saved.

**Program B:** If Program B is adopted, there is 5% probability that 600 people will be saved and 95% probability that no people will be saved.

You are working in a laboratory which is inventing a medicine that will help to stop the spread of HIV. As a result of conducting an experiment with the new medicine, 600 people might die. You have come up with two alternative programs to lessen the harm caused by the medicines. Choose one of the following programs:

**Program A:** If Program A is adopted, 570 people will die.

**Program B:** If Program B is adopted, there is 5% probability that no people will die and 95% probability that 600 people will die.

You are working in a laboratory which is inventing a medicine that will help to stop the spread of HIV. As a result of conducting an experiment with the new medicine, 600 people might die. You have come up with two alternative programs to lessen the harm caused by the medicines. Choose one of the following programs:

**Program A:** If Program A is adopted, some people represented in black will be saved (see the pie-chart below).

**Program B:** If Program B is adopted, the probability that 600 people will be saved is represented in black and the probability that no people will be saved is represented in grey (see the pie-chart below).

You are working in a laboratory which is inventing a medicine that will help to stop the spread of HIV. As a result of conducting an experiment with the new medicine, 600 people might die. You

have come up with two alternative programs to lessen the harm caused by the medicines. Choose one of the following programs:

**Program A:** If Program A is adopted, some people represented in grey will die (see the pie-chart below).

**Program B:** If Program B is adopted, the probability that no people will die is represented in black and the probability that 600 people will die is represented in grey (see the pie-chart below).

You are working in a laboratory which is inventing a medicine that will help to stop the spread of HIV. As a result of conducting an experiment with the new medicine, 600 people might die. You

have come up with two alternative programs to lessen the harm caused by the medicines. Choose one of the following programs:

**Program A:** If Program A is adopted, some people represented in black will be saved (see the pie-chart below).

**Program B:** If Program B is adopted, the probability that 600 people will be saved is represented in black and the probability that no people will be saved is represented in grey (see the pie-chart below).

You are working in a laboratory which is inventing a medicine that will help to stop the spread of HIV. As a result of conducting an experiment with the new medicine, 600 people might die. You

have come up with two alternative programs to lessen the harm caused by the medicines. Choose one of the following programs:

**Program A:** If Program A is adopted, some people represented in grey will die (see the pie-chart below).

**Program B:** If Program B is adopted, the probability that no people will die is represented in black and the probability that 600 people will die is represented in grey (see the pie-chart below).

*Finance domain*

You are working for a financial institution and there are some stocks you have invested in have started fluctuating in price. As a result, you might lose ₤600.You have come up with two alternative programs to lessen the harm caused by the fluctuation. Choose one of the following programs:

**Program A:** If Program A is adopted, ₤200 will be saved.

**Program B:** If Program B is adopted, there is 33.3% probability that ₤600 will be saved and 66.7% probability that no money will be saved.

You are working for a financial institution and there are some stocks you have invested in have started fluctuating in price. As a result, you might lose ₤600.You have come up with two alternative programs to lessen the harm caused by the fluctuation. Choose one of the following programs:

**Program A:** If Program A is adopted, ₤400 will be lost.

**Program B:** If Program B is adopted, there is 33.3% probability that no money will be lost and 66.7% probability that ₤600 will be lost.

You are working for a financial institution and there are some stocks you have invested in have started fluctuating in price. As a result, you might lose ₤600. You have come up with two alternative programs to lessen the harm caused by the fluctuation. Choose one of the following programs:

**Program A:** If Program A is adopted, ₤30 will be saved.

**Program B:** If Program B is adopted, there is 5% probability that ₤600 will be saved and 95% probability that no money will be saved.

You are working for a financial institution and there are some stocks you have invested in have started fluctuating in price. As a result, you might lose ₤600. You have come up with two alternative programs to lessen the harm caused by the fluctuation. Choose one of the following programs:

**Program A:** If Program A is adopted, ₤570 will be lost.

**Program B:** If Program B is adopted, there is 5% probability that no money will be lost and 95% probability that ₤600 will be lost.

You are working for a financial institution and there are some stocks you have invested in have started fluctuating in price. As a result, you might lose ₤600. You have come up with two alternative programs to lessen the harm caused by the fluctuation. Choose one of the following programs:

**Program A:** If Program A is adopted, some money represented in black will be saved (see the pie-chart below).

**Program B:** If Program B is adopted, the probability that ₤600 will be saved is represented in black and the probability that no money will be saved is represented in grey (see the pie-chart below).

You are working for a financial institution and there are some stocks you have invested in have started fluctuating in price. As a result, you might lose ₤600. You have come up with two alternative programs to lessen the harm caused by the fluctuation. Choose one of the following programs:

**Program A:** If Program A is adopted, some money represented in grey will be lost (see the pie-chart below).

**Program B:** If Program B is adopted, the probability that no money will be lost is represented in black and the probability that ₤600 will be lost is represented in grey (see the pie-chart below).

You are working for a financial institution and there are some stocks you have invested in have started fluctuating in price. As a result, you might lose ₤600. You have come up with two alternative programs to lessen the harm caused by the fluctuation. Choose one of the following programs:

**Program A:** If Program A is adopted, some money represented in black will be saved (see the pie-chart below).

**Program B:** If Program B is adopted, the probability that ₤600 will be saved is represented in black and the probability that no money will be saved is represented in grey (see the pie-chart below).

You are working for a financial institution and there are some stocks you have invested in have started fluctuating in price. As a result, you might lose ₤600. You have come up with two alternative programs to lessen the harm caused by the fluctuation. Choose one of the following programs:

**Program A:** If Program A is adopted, some money represented in grey will be lost (see the pie-chart below).

**Program B:** If Program B is adopted, the probability that no money will be lost is represented in black and the probability that ₤600 will be lost is represented in grey (see the pie-chart below).

*Technology domain*

You are working for a factory which specializes in the manufacturing of mobile phones and your main engineer suggested implementing new features to the phones. As a result, 600 mobile phones might be damaged. You have come up with two alternative programs to lessen the harm of the damages. Choose one of the following programs:

**Program A:** If Program A is adopted, 200 mobile phones will be saved.

**Program B:** If Program B is adopted, there is 33.3% probability that 600 mobile phones will be saved and 66.7% probability that no mobile phones will be saved.

You are working for a factory which specializes in the manufacturing of mobile phones and your main engineer suggested implementing new features to the phones. As a result, 600 mobile phones might be damaged. You have come up with two alternative programs to lessen the harm of the damages. Choose one of the following programs:

**Program A:** If Program A is adopted, 400 mobile phones will be damaged.

**Program B:** If Program B is adopted, there is 33.3% probability that no mobile phones will be damaged and 66.7% probability that 600 mobile phones will be damaged.

You are working for a factory which specializes in the manufacturing of mobile phones and your main engineer suggested implementing new features to the mobile phones. As a result, 600 mobile phones might be damaged. You have come up with two alternative programs to lessen the harm caused by the implementation. Choose one of the following programs:

**Program A:** If Program A is adopted, 30 mobile phones will be saved.

**Program B:** If Program B is adopted, there is 5% probability that 600 mobile phones will be saved and 95% probability that no mobile phones will be saved.

You are working   for a factory which specializes in the manufacturing of mobile phones and your main engineer suggested implementing new features to the mobile phones. As a result, 600 mobile phones might be damaged. You have come up with two alternative programs to lessen the harm caused by the implementation. Choose one of the following programs:

**Program A:** If Program A is adopted, 570 mobile phones will be damaged.

**Program B:** If Program B is adopted, there is 5% probability that no mobile phones will be damaged and 95% probability that 600 mobile phones will be damaged.

You are working   for a factory which specializes in the manufacturing of mobile phones and your main engineer suggested implementing new features to the mobile phones. As a result, 600 mobile phones might be damaged. You have come up with two alternative programs to lessen the harm caused by the implementation. Choose one of the following programs:

**Program A:** If Program A is adopted, some mobile phones represented in black will be saved (see the pie-chart below).

**Program B:** If Program B is adopted, the probability that 600 mobile phones will be saved is represented in black and the probability that no mobile phones will be saved is represented in grey (see the pie-chart below).

You are working for a factory which specializes in the manufacturing of mobile phones and your main engineer suggested implementing new features to the mobile phones. As a result, 600 mobile phones might be damaged. You have come up with two alternative programs to lessen the harm caused by the implementation. Choose one of the following programs:

**Program A:** If Program A is adopted, some mobile phones represented in grey will be damaged (see the pie-chart below).

**Program B:** If Program B is adopted, the probability that no mobile phones will be damaged is represented in black and the probability that 600 mobile phones will be damaged is represented in grey (see the pie-chart below).

You are working for a factory which specializes in the manufacturing of mobile phones. Your main engineer suggested implementing new features to the mobile phones. As a result, 600 mobile phones might be damaged. You have come up with two alternative programs to lessen the harm caused by the implementation. Choose one of the following programs:

**Program A:** If Program A is adopted, some mobile phones represented in black will be saved (see the pie-chart below).

**Program B:** If Program B is adopted, the probability that 600 mobile phones will be saved is represented in black and the probability that no mobile phones will be saved is represented in grey (see the pie-chart below).

You are working for a factory which specializes in the manufacturing of mobile phones. Your main engineer suggested implementing new features to the mobile phones. As a result, 600 mobile phones might be damaged. You have come up with two alternative programs to lessen the harm caused by the implementation. Choose one of the following programs:

**Program A:** If Program A is adopted, some mobile phones represented in grey will be damaged (see the pie-chart below).

**Program B:** If Program B is adopted, the probability that no mobile phones will be damaged is represented in black and the probability that 600 mobile phones will be damaged is represented in grey (see the pie-chart below).

1. **List of occupations categorized into two types of occupational background**

| “White-collar” occupations (*N*=60) | “High-risk” occupations (*N*=60) |
| --- | --- |
| Financial advisor | Pharmacist |
| Financial analyst | Nurse |
| Business analyst | Ambulance worker |
| Clerk | Surgeon |
| General store manager | Support worker |
| Derivatives dealer | Police officer |
| Administrator | Public transport driver |
| IT consultant | Ambulance driver |
| Account manager | Constructions worker |
| Business manager | Maintenance worker |
| Retail sales assistant | Fire-fighter |
